# Impact of antiphospholipid antibodies on thrombotic events in ambulatory cancer patients

**DOI:** 10.1371/journal.pone.0279450

**Published:** 2023-01-20

**Authors:** Chalermkiat Kansuttiviwat, Piangrawee Niprapan, Adisak Tantiworawit, Lalita Norasetthada, Ekarat Rattarittamrong, Thanawat Rattanathammethee, Sasinee Hantrakool, Pokpong Piriyakhuntorn, Teerachat Punnachet, Nonthakorn Hantrakun, Chatree Chai-adisaksopha

**Affiliations:** 1 Department of Internal Medicine, Faculty of Medicine, Chiang Mai University, Chiang Mai, Thailand; 2 Division of Hematology, Department of Internal Medicine, Faculty of Medicine, Chiang Mai University, Chiang Mai, Thailand; Ataturk University Faculty of Medicine, TURKEY

## Abstract

**Background:**

Despite the conflicting data, the positivity of antiphospholipid antibodies (aPL) in cancer patients may be associated with an increased risk of thrombosis.

**Objective:**

To identify the prevalence and impact of aPL on venous thromboembolic events (VTE) and arterial thrombosis (ATE) in ambulatory cancer patients

**Methods:**

In this single-center, prospective cohort study, we enrolled newly diagnosed ambulatory cancer patients receiving chemotherapy. Non-cancer controls were age- and sex-matched. Participants were evaluated for aPL. Primary outcomes were the composite outcome of VTE or ATE and the prevalence of aPL positivity in cancer patients. Secondary outcomes included the risk of VTE and ATE in cancer patients and all-cause mortality at six-month follow-up duration.

**Results:**

There were 137 cases and 137 controls with mean age of 56.0±12.3 and 55.5±12.1 years, respectively. Cancer patients were more likely to have positive aPL compared to controls, with the risk difference of 9.4% (95%CI 1.5 to 17.5). Composite of ATE or VTE occurred in 9 (6.6%) in cancer patients and 2 (1.5%) in controls. Cancer patients with aPL positivity were associated with higher risk of ATE or VTE (risk ratio [RR] 3.6, 95% CI 1.04–12.4). Positive LA in cancer patients were associated with higher risk of composites of ATE or VTE (RR 5.3 95%CI 1.3–21.0), whereas the anti-β2-GPI positivity were associated with increased risk of VTE (RR 4.7, 95%CI 1.1–19.2).

**Conclusion:**

aPL was more prevalent in active cancer patients and positive aPL in cancer patients was associated with arterial or venous thrombosis.

## Introduction

Thrombotic events, both venous thromboembolism (VTE) and arterial thromboembolism (ATE), remain one of the major complications in cancer patients and contribute to the second-leading cause of mortality [1]. The pathogenesis of cancer-associated thrombosis mainly involves a prothrombotic state [2–5]. Cancer patients tend to have an approximately 5- to 7-fold increased risk of developing venous thromboembolism (VTE) [2, 3] Cancer patients with VTE have a significantly worse prognosis than those without VTE [4].

Among several mechanisms of hypercoagulability state, the antiphospholipid antibodies (aPL) are considered one of the possible mechanisms for promoting clots in cancer patients. aPL is a group of autoantibodies that interacts with self-proteins on the endothelial cells [6]. The aPL-mediated pathogenesis causes inflammation of blood vessels and promotes coagulation, resulting in an increased risk of thrombosis [6, 7]. Three of the most common detectable autoantibodies are lupus anticoagulant (LA), anti-cardiolipin antibodies (aCL), and anti-β2-glycoprotein-I antibodies (aβ2GPI) [6]. The aPL positivity is considered clinically significant if the aPL positivity is confirmed on two occasions at least 12 weeks apart [7, 8]. Generally, aPL positivity is included as diagnostic criteria for antiphospholipid syndrome [9]. Several studies demonstrated the association between the positivity of aPL and cancer [10–18].

Due to the absence of population-based studies, the true prevalence of aPL positivity in the general population is still unknown. A prospective study of healthy blood donors demonstrated that 10% of healthy blood donors were positive for aCL, and 1% were positive for LA. Moreover, after being followed for one year, less than 1% of these donors still had positive aPL [19]. The prevalence of aPL positivity in cancer patients varies widely between 1.4% to 74% [10–18].

Currently, the roles of aPL in the promotion of thrombosis in cancer patients remain poorly investigated. The thrombosis is proposed to be mediated by the immune system as a response to tumor antigens, cancer immunotherapy, or systemic inflammation from malignancies [7, 8, 10, 13–15, 17].^.^ Furthermore, cancers are found to be associated with catastrophic antiphospholipid syndrome (CAPS), a condition that is fatal and leads to the rapid development of thrombosis in multiple organs. The analysis from the international CAPS registry found that 16% of included 500 CAPS patients have cancer, mostly lymphomas, and leukemias [20, 21].

No well-designed studies are currently available to determine the prevalence of anti-phospholipid antibodies and their effect on the incidence of VTE and ATE in cancer patients. In this study, we determined to identify the prevalence of aPL positivity in ambulatory cancer patients, compared to healthy subjects in a similar age group. We also aimed to compare thrombotic events between cancer patients with aPL positivity with those with negative results

## Material and methods

### Study design and setting

This was a single-center, prospective cohort study conducted at Maharaj Nakorn Chiang Mai Hospital, Faculty of Medicine, Chiang Mai University, Chiang Mai, Thailand. This study was approved by the Research Ethics Committee of Faculty of Medicine, Chiang Mai University. All included patients and controls were included in this study from July 2021 to July 2022. The follow-up period for clinical outcomes was six months after the initial inclusion.

### Participants

Consecutive ambulatory cancer patients were enrolled if they met the inclusion criteria for the cancer group, including (1) age between 18–80 years, (2) newly diagnosed or recently confirmed relapse of either solid or hematologic malignancies, and (3) planned to receive chemotherapy at one-day ambulatory chemotherapy unit.

In the control group, we enrolled age- and sex-matched healthy participants who visited the blood bank unit for blood donation or visited outpatient department for annual health checkups.

Participants in both groups were excluded if any of the following condition(s) 1) prior history of autoimmune diseases or antiphospholipid syndrome or confirmed positive aPL before entry of this study, 2) currently taking anticoagulants (including warfarin, heparin, or direct oral anticoagulants) within 6 months before inclusion of this study and 3) active arterial or venous thrombosis within 3 months prior to enrollment.

### Laboratory evaluation

Participants in both groups were tested for aPL upon the entry of this study, including LA, aCL IgM, and IgG and aβ2GPI IgM and IgG. A total of 13 mL of blood was collected and was processed via double-spin centrifugation at room temperature (25°C) before aPL testing. A 3-mL clotted venous blood was collected for LA testing in two separate methods, the HemosIL^®^ silica clotting time (SCT) method and the HemosIL^®^ dRVVT method. The cut-off threshold for LA ratio was 1.27 for SCT and 1.27 for dRVVT. The aCL and aβ2GPI IgM and IgG testing were tested using the Euroimmun^®^ ELISA kit test, using 5-mL clotted venous blood for each aPL test. The cut-off limit for aCL and aβ2GPI were 40 relative units/mL, respectively [6, 9, 22]. The aPL testing were repeated in participants with initial aPL positivity at the interval of at least 12 weeks apart from the initial testing.

### Outcomes

The participants in both groups were followed for 6 months after enrolling in the study. The primary outcome was the composite outcomes of ATE or VTE, defined as any thrombotic events either from arterial sites or venous sites that occurred within 6 months of the study period, and the prevalence of aPL positivity in cancer patients and non-cancer healthy participants. Secondary outcomes were the incidence of ATE, incidence of VTE, and all-cause mortality up to six months follow-up period. The data regarding to the thrombotic outcomes was obtained from the official report of radiologic evidence, including venous ultrasonography, computerized tomography (CT) of chest or abdomen, or conventional angiography (i.e. coronary angiogram) as clinically indicated if available during the 6-month follow-up period. 6-month mortality was reviewed from the electronic medical record.

### Statistical analysis

The sample size was calculated based on the hypothesis that the incidence of thrombotic events in cancer patients with positive aPL was 8-fold higher than those with negative aPL (8% VS 1%) [17]. Therefore, the sample size of 137 participants per group was obtained to achieve the power of 80% and 5% of type 1 error.

Data obtained in this study were analyzed with STATA/MP version 14.0. The characteristics of the cohort and control, including types of malignancies and the antiphospholipid profiles in both groups, were described with descriptive statistics. The composite outcomes of ATE or VTE and 6-month mortality are analyzed with logistic regression and reported an odds ratio (OR), and risk ratio (RR) with a 95% confidence interval (CI). P-value < 0.05 was considered statistically significant for all analyses.

## Results and discussion

### Baseline characteristics

A total number of 274 participants were included (137 in cancer cohort and 137 in control cohort). The baseline characteristics from each group are shown in Table 1. Participants in cancer cohort were more likely to have cirrhosis, history of smoking, and history of alcohol consumption. There was a significantly higher proportion of cancer patients receiving recent surgery within 6 months compared to those in control cohort. The laboratory profiles, except for anemia, were not statistically different among groups.

**Table 1:** Baseline characteristics of the cancer patients and control in this study.

Baseline Characteristics	Cancer patients (n = 137)	Control (n = 137)	p-value
Age (years)	56.0 ± 12.3	55.5 ± 12.1	0.77
Male (%)	50.4% (69/137)	51.1% (70/137)	0.90
Weight (kg)	57.0 ± 10.5	62.5 ± 11.6	0.35
Height (cm)	160.1 ± 8.3	160.3 ± 8.3	0.39
BMI (kg/m^2^)	22.2 ± 3.3	24.2 ± 3.3	0.54
Underlying Diseases			
Hypertension	32.9% (45/137)	36.5% (50/137)	0.52
Diabetes	11.7% (16/137)	19.0% (26/137)	0.09
Dyslipidemia	18.3% (25/137)	26.3% (36/137)	0.11
Stroke/TIA	0.0% (0/137)	1.4% (2/137)	0.16
Cirrhosis	9.5% (13/137)	0.0% (0/137)	<0.001
Previous VTE	1.5% (2/137)	0.0% (0/137)	0.16
Smoking	48.2% (66/137)	2.9% (4/137)	<0.001
Alcohol drinking	33.6% (46/137)	20.4% (28/137)	0.01
Recent surgery within 6 months	29.9% (41/137)	0.0% (0/137)	<0.001
Laboratory investigations			
Hemoglobin (g/dL)	11.7 ± 2.3	13.6 ± 1.4	0.01
WBC count (x 10^9^ cells/L)	7.9 ± 4.2	6.8 ± 2.0	0.42
Platelet (x10^9^ cells/L)	283.7 ± 121.9	271.2 + 59.9	0.32
BUN (mg/dL)	12.8 ± 6.1	14.2 ± 5.6	0.19
Creatinine (mg/dL)	1.0 ± 1.8	0.9 ± 0.3	0.62
Estimated GFR (mL/min/1.73 m^2^)	90.0 ± 21.6	86.8 ± 20.4	0.50
AST (U/L)	36.6 ± 35.8	23.7 ± 14.3	0.61
ALT (U/L)	30.9 ± 47.5	21.6 ± 12.1	0.28
ALP (U/L)	110.3 ± 71.0	71.3 ± 22.0	0.45
Total bilirubin (mg/dL)	0.6 ± 0.6	0.7 ± 0.5	0.12
Albumin (g/dL)	4.0 ± 0.6	4.3 ± 0.4	0.31
PT (sec)	12.1 ± 1.5	11.1 ± 1.1	0.51
PTT (sec)	31.5 ± 5.0	30.9 ± 4.1	0.10

Abbreviations: AST = Aspartate aminotransferase, ALT = Alanine aminotransferase, ALP = Alkaline phosphatase, PT = Prothrombin time PTT = Partial thromboplastin time, TIA = Transient ischemic attack, VTE = thromboembolic events, WBC = White blood cells

In cancer cohort, majority of the patients were diagnosed solid tumors (85.4%), Table 2. The most prevalent site of cancer was gynecologic, followed by hepatobiliary tract and gastrointestinal cancer, with the proportion of 23.4%, 18.3% and 16.0%, respectively. Hematologic malignancies were accounted for 14.6% of remaining cancer patients.

**Table 2:** Types of malignancy included in this study.

Types of malignancy (n = 137)	Frequency (%)
Hematologic malignancy	20/137 (14.6%)
Lymphoma	14/137 (10.2%)
Leukemia	6/137 (4.4%)
Solid malignancy	117/137 (85.4%)
Gynecologic	32/137 (23.4%)
Hepatobiliary	25/137 (18.3%)
Gastrointestinal	22/137 (16.1%)
Thoracic (lung)	10/137 (7.3%)
Urologic	7/137 (5.1%)
Breast	1/137 (0.7%)
Head & neck	5/137 (3.7%)
Other soft tissue tumors	15/137 (11.0%)

### Antiphospholipid profiles

**Fig 1:**
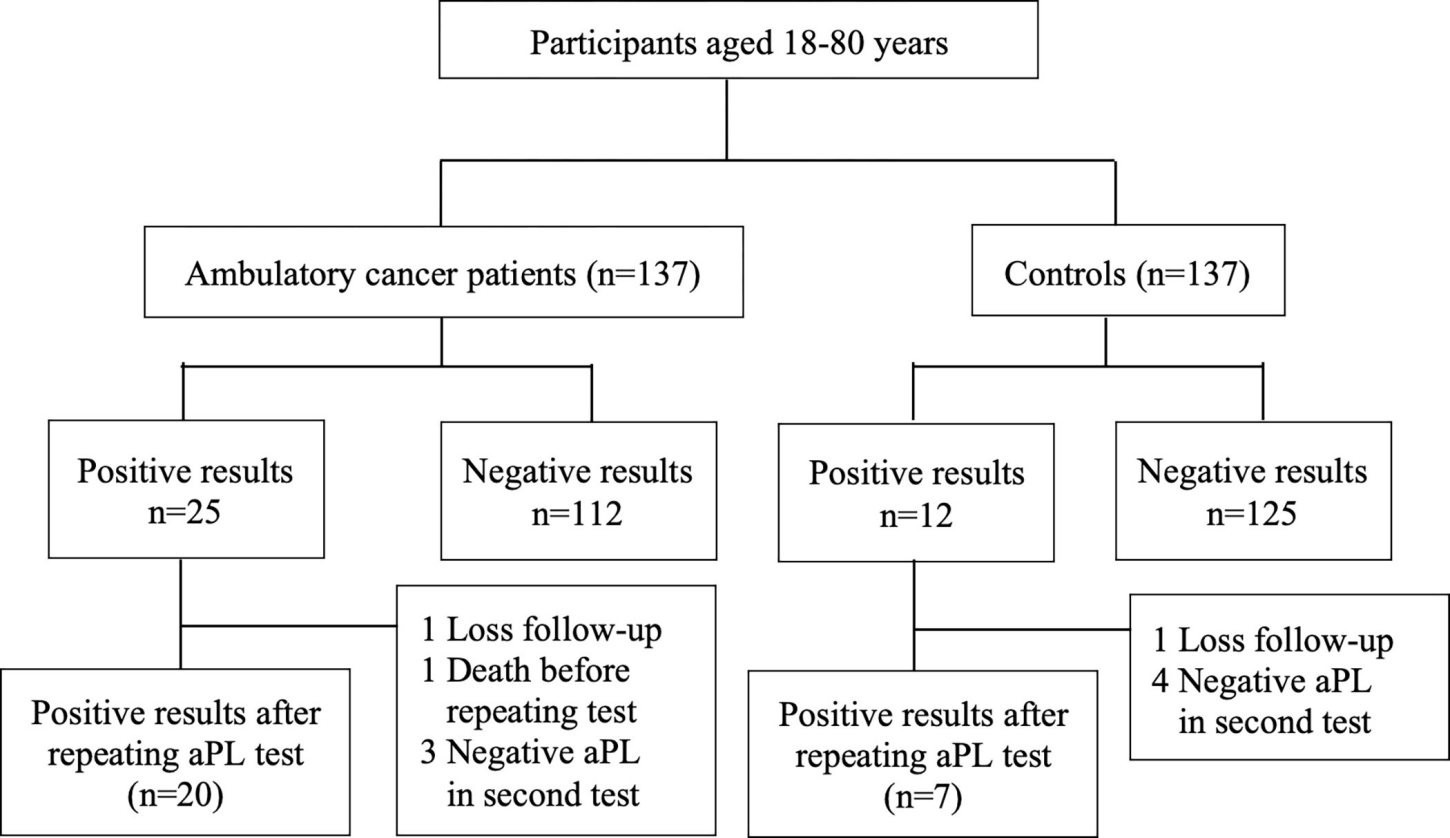
Flow diagram of participants in the study.

**Fig 2:**
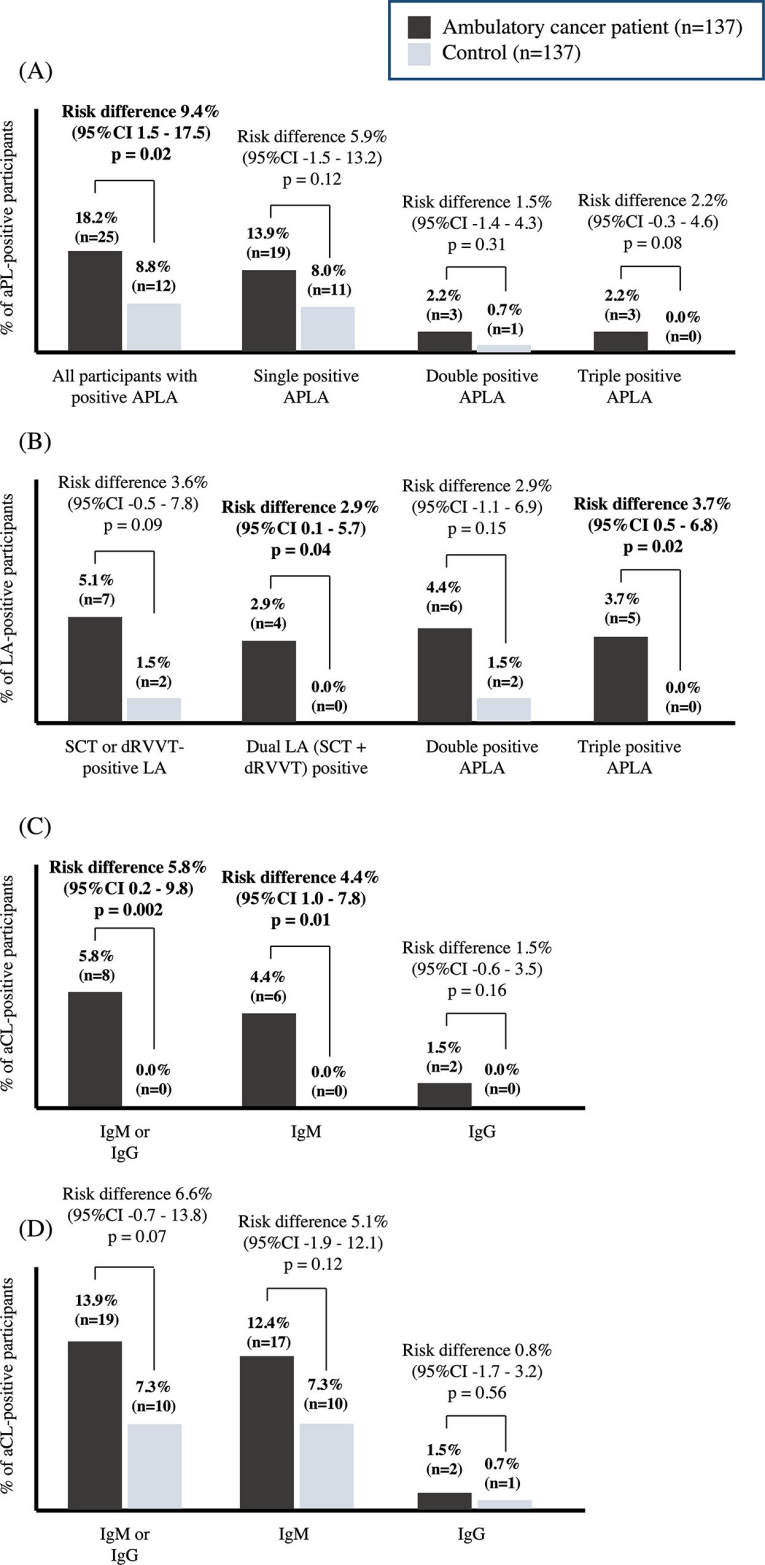
aPL profile in cohort and control groups (A) according to number of positive aPL, (B) LA-positive, (C) aCL-positive, and (D) aβ2-GPI-positive participants. Abbreviations: aPL = antiphospholipid antibodies, aCL = anti-cardiolipin, aβ2GPI = anti-beta-2 glycoprotein I, LA = lupus anticoagulant, SCT = silica clotting time, dRVVT = diluted Russell’s Viper Venom time.

The flow diagram in Fig 1 depicts the numbers of subjects with positive aPL at initial blood testing and participants with positive aPL after a second blood test. A total of 25 cancer patients (18.2%) and 12 participants from control group (8.8%) had a positive result for any aPL testing. Overall, cancer patients had higher numbers of any positive aPL with a risk difference of 9.4% (95% CI 1.5 to 17.5), P = 0.02 (Fig 2) and relative risk (RR) of 2.08 (95%CI 1.09–3.98), P = 0.02 After repeating aPL testing at 12 weeks, 20 (14.6%) ambulatory cancer patients were still positive for aPL, and 7 (5.1%) participants from the control group had positive aPL results.

Titers of aPL (aCL-IgM, aCL-IgG, anti-β2-GPI IgM and anti-β2-GPI IgG) is shown in Table 3. The medians of aCL-IgM, aCL-IgG, anti-β2-GPI IgM and anti-β2-GPI IgG were significantly higher among cancer patients as compared to controls.

**Table 3:** Titers of antiphospholipid antibodies (aCL IgG and IgM and β2GPI-IgG and IgM) in cancer patients VS controls.

Antibodies	Mean (SD)	Median (IQR)	p-value
**aCL-IgM (U/mL)**			
Cancer (n = 137)	5.1 (11.7)	2 (2, 2.7)	0.04
Control (n = 137)	3.5 (10.2)	2 (2, 2.3)	
**aCL-IgG (U/mL)**			
Cancer (n = 137)	3.6 (9.7)	2 (2, 2.1)	0.003
Control (n = 137)	2.2 (1.0)	2 (2, 2)	
**aβ2GPI-IgM (U/mL)**			
Cancer (n = 137)	13.5 (25.4)	5.4 (2.3, 13.3)	0.005
Control (n = 137)	9.9 (22.3)	3.5 (0.2, 9.9)	
**aβ2GPI-IgG (U/mL)**			
Cancer (n = 137)	4.3 (7.9)	2.6 (2.1, 4.2)	0.002
Control (n = 137)	2.8 (4.6)	2.2 (0, 3.1)	

Abbreviations: aCL; anticartiolipin, aβ2GPI; anti-β2 glycoprotein I, U/mL; relative units/millimeter, SD; standard deviation, IQR; interquartile range

The antiphospholipid profiles are summarized in Fig 2. Cancer patients were more likely to have dual LA or single dRVVT positivity as compared to controls with a risk difference of 5.8% (95% CI 0.2 to 9.7), P = 0.04 and 3.7% (0.5–6.8), P = 0.02, respectively. Moreover, cancer patients were more likely to have aCL positivity (IgG or IgM), comparing to control with a risk difference of 5.8% (95% CI 0.2 to 9.7), P = 0.002. However, cancer patients had numerical higher in terms of an anti-β2-GPI IgM or IgG positivity when compared to control with a risk difference of 6.6% (-0.7 to 13.1, P = 0.07).

### Thromboembolic events

The summary of outcomes is shown in Table 4. Cancer patients were associated with significantly higher risk of composite outcomes of ATE or VTE comparing with controls, 6.6% versus 1.5% (OR 4.7, 95%CI 0.9–45.7, P = 0.03).

**Table 4:** Comparison of outcomes in cohort and control groups.

Outcome	Patients	Control	Odds Ratio	P-value
	(n = 137)	(n = 137)	(95%CI)	
Composite outcome of VTE and ATE	9/137 (6.6%)	2/137 (1.5%)	4.8 (1.0–45.7)	0.03
VTE	7/137 (5.8%)	0/137	15.8 (0.9–279.5)	0.06*
- Deep vein thrombosis	6/137 (4.4%)	0/137	13.6 (0.8–243.7)	0.07*
- Pulmonary embolism	1/137 (0.7%)	0/137	3.0 (0.1–74.8)	0.50*
- Other sites of VTE	2/137 (1.5%)	0/137	5.1 (0.2–106.7)	0.29*
ATE	2/137 (1.5%)	2/137 (1.5%)	1.0 (0.1–14.0)	1.00
Death	19/137 (13.9%)	0/137	45.3 (2.7–757.6)	0.008*

* Zero cell count: calculates odd ratios and significance tests by using Firth’s penalized logistic regression

Abbreviations: ATE = arterial thrombotic events, VTE = Venous thromboembolic events

**Fig 3:**
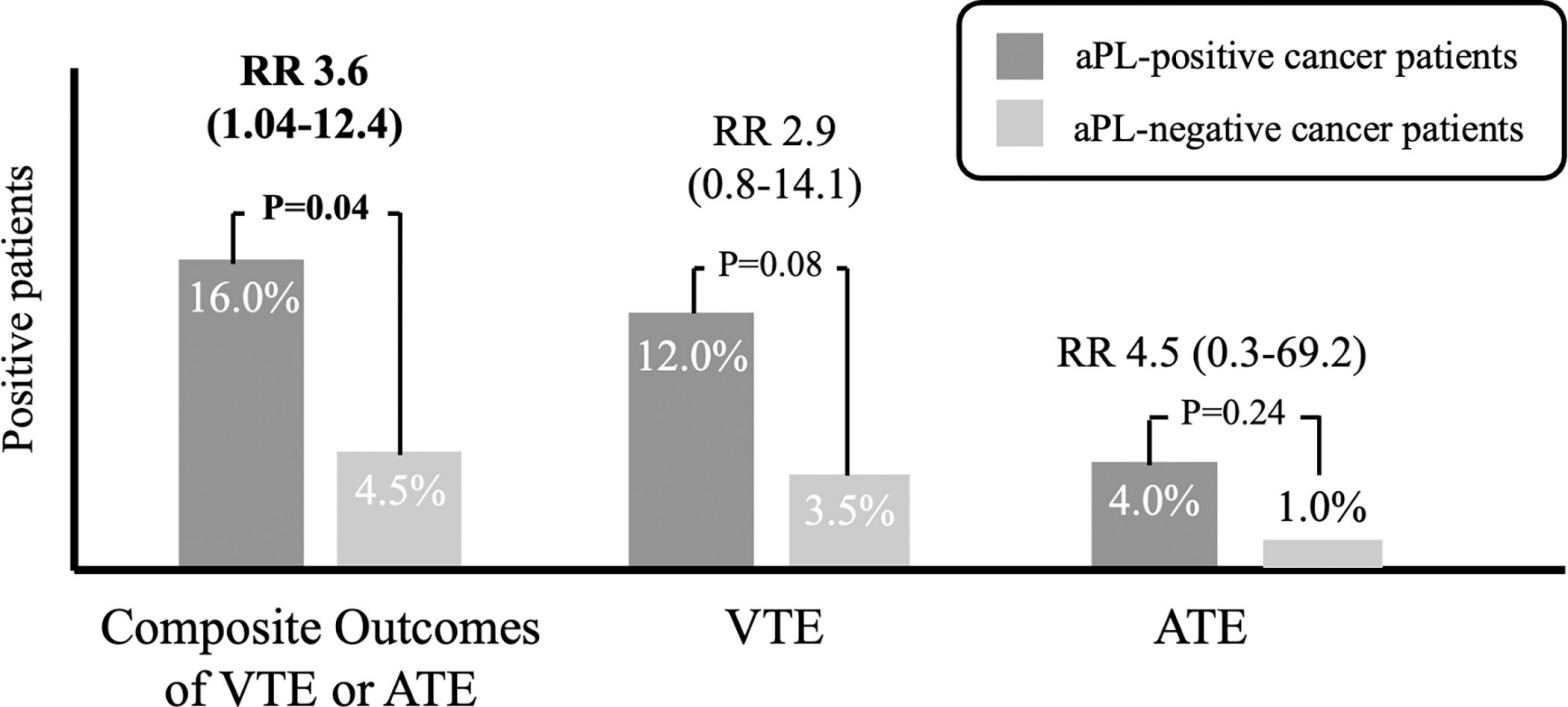
Composite outcomes of VTE or ATE, VTE and ATE in aPL-positive and aPL-negative cancer patients. Abbreviations: ATE = arterial thrombotic events, VTE = venous thromboembolic events, RR = relative risk.

**Fig 4:**
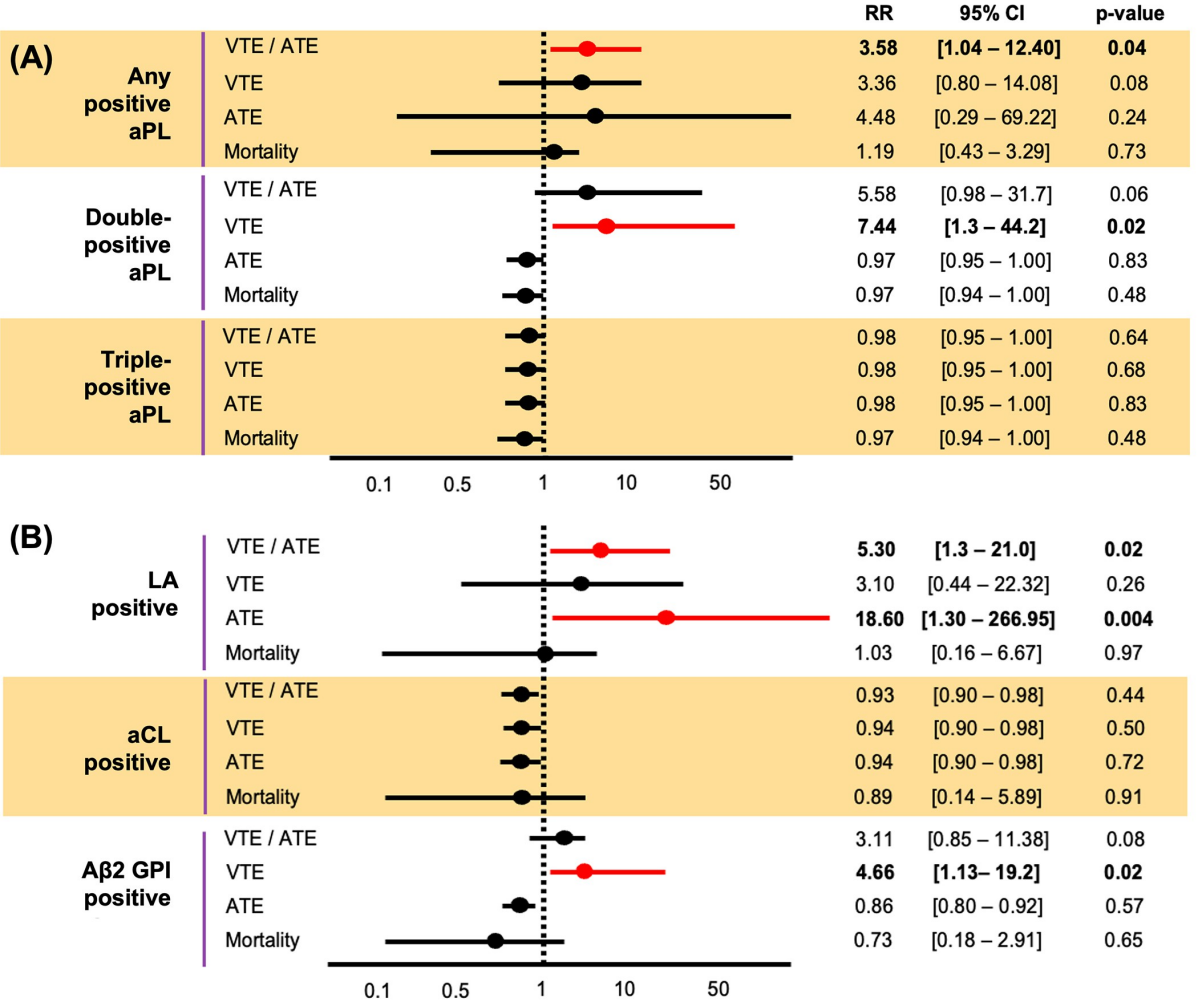
(A) Association of thrombotic outcomes and numbers of positive aPL, and (B) types of positive aPL status in ambulatory cancer patients. Abbreviations: ATE = arterial thrombotic events, aCL = anticardiolipin, LA = lupus anticoagulant, aβ2GPI = anti-beta-2 glycoprotein I.

Figs 3 and 4 demonstrate the association of the presence of aPL and ATE, VTE or mortality in cancer patients. A total 16.0% of cancer patients with any positive aPL developed composites of ATE or VTE when compared with 4.5% in those with negative aPL. Any aPL positivity in cancer patients were associated with higher risk of composites, with RR of 3.6, 95%CI 1.04–12.4, p = 0.04. There were higher proportions of any aPL-positive cancer patients with VTE (12.0%) than aPL-negative cancer patients (3.5%). The difference, however, was not statistically significant with RR 2.9, 95%CI 0.8–14.1, p = 0.08. The difference in ATE was also insignificant between both groups, as shown in Fig 3.

Cancer patients with double positive aPL were associated with higher risk of VTE with RR of 7.4, 95%CI 1.3–44.2, p = 0.02. We did not observe an increased risk of ATE or VTE in cancer patients who had triple positive aPL as compared to those with negative aPL, RR 0.98, 95%CI 0.95–1.00, p = 0.64 (Fig 4).

When classified by type of aPL, cancer patients who had positive LA were associated with significantly increased risk of composites outcome of ATE or VTE with RR of 5.3, 95%CI 1.3–21.0, p = 0.02 and ATE with RR of 18.6, 95%CI 1.3–267.0, p = 0.004. Cancer patients with aβ2GPI positivity were associated with significant higher risk of VTE as compared to those with negative results, RR 4.7 (95%CI 1.13–19.20), p = 0.02. Cancer patients who had positive ACL had comparable risk of VTE or ATE as compared to those with test negative.

### 6-month mortality

All-cause mortality at 6 months of follow-up period was observed in 13.9% of cancer patients and 0% of controls (OR 42.2, 95%CI 2.7–757.6, p = 0.008), as demonstrated in Table 3. Most patients died from infectious causes or progressive cancer disease. Within the cancer patients, there were no significant differences in all-cause mortality between aPL-positive cancer patients and those with negative antibodies, as shown in Fig 4.

## Discussion

In this study, we found that cancer patients were approximately 2-time more likely to have aPL positivity as compared to age- and sex-matched non-cancer participants (18.2% VS 8.8%). The prevalence of aPL positivity from our study correlates within the ranges of prevalence demonstrated in the previous studies, which reported the prevalence of 10%-24% [11–18].

ATE and VTE were important outcomes associated with APS. Moreover, thrombotic events (either ATE or VTE) were defined as a clinical criterion for the diagnosis of APS [6, 22]. Therefore, our study focused on the occurrence of both ATE and VTE as primary outcome. In this study, the composite outcomes of ATE or VTE occurred 4-fold higher in cancer patients as compared with controls. The risk of developing VTE were higher in cancer group than in control group (5.8% vs 0%). Whereas the risk of ATE were comparable between cancer patients and controls (1.5% vs 1.5%). The differences between two groups were not statistically significant when analyzed in each category of thrombotic events. It could be explained from inadequate power to determine the difference in VTE or ATE individually, along with lower rates of VTE in Asian populations. The finding in this study was comparable to the previous studies. The relative risk of VTE in cancer patients compared with non-cancer population are ranging from 4 to 7 [2, 3, 23]. In Asian populations, the risk of developing VTE in cancer patients is approximately 1.6- to 2-fold higher compared to general population [24–26]. Since the rate of thrombotic complications in this current study is comparable to previous reports, this confirmed the validity of the cohort.

In addition, cancer patients who exhibited aPL positivity in this study had a 3.6-fold increased risk of developing VTE or ATE compared to cancer patients whose tests were negative. Moreover, cancer patients with double-positive aPL also had a 7.4-fold higher relative risk of developing VTE.

The association between cancer and antiphospholipid lipid antibodies has been investigated in several studies. The Italian study reported that cancer patients had a 6-time higher risk of low titer aPL positive compared to control participants who did not have cancer [16]. However, the study did not observe a statistical difference in thrombosis between aPL-positive and aPL-negative patients. On the contrary, a matched control study reported that cancer patients with thrombosis had a higher prevalence of aPL positivity (8.0%), compared to cancer patients without thrombosis (1.4%) and healthy controls (0.4%) [17]. The variation in the difference of the prevalence of aPL and thrombotic outcomes might be explained by the definition of aPL positivity, study design, the population of cancer patients, and the selection of control. Our study was a prospective cohort, in which we followed all participants for 6 months. The definition of aPL positivity was diagnosed based on international recommendation [6, 22]. All patients who had positive results were invited to have a repeated test. The cases and controls were well-matched based on age and sex, which were the variables associated with aPL positivity [27–29]. We hypothesized that aPL may be one of the possible pathogenic mechanisms for thrombotic complications in cancer patients.

Although, triple aPL positivity was associated with an increased risk of thrombosis in non-cancer patients in previous studies [30–32]. In this current study, the number of patients with triple-positive antibodies was extremely low. Consequently, this study was unpowered to detect any differences in the thrombotic outcomes among patients who had triple aPL positivity. There was no statistical difference in 6-month mortality between aPL-positive and aPL-negative patients observed in this study.

When classified by isotype of aPL, cancer patients in this current study had a higher proportion of test positivity when compared to controls for every isotype (LA (5.1% vs 1.5%), aCL (5.8% vs 0.0%) and aβ2GPI (13.9% vs 7.3%). The distribution of aPL subtypes in active cancer patients remains inconclusive. Several studies reported various subtypes of aPL found predominantly in cancer patients [11, 13, 18]. A recent systematic review reported that patients with gastrointestinal cancer, genitourinary tract cancer, and lung cancer had a high risk of having aCL positivity. However, one of the limitations of this review was most studies did not report isotype distribution or measured only 1 isotype [8]. Based on the recent evidence, it was difficult to conclude the association between cancer and the occurrence of isotype of aPL.

aPL is one of the currently well-known acquired immune-mediated causes for thrombo-embolism. However, the exact pathogenesis of which how the aPL is produced in cancer patients is still unknown. aPL could be produced as a humoral response against tumor antigens [7]. It was postulated that the accumulation of cancer cells may trigger the overproduction of aPL by excessive proliferation and inadequate apoptosis of cancer cells. [8]. The exposure of phosphatidylserine on the outer membrane during the apoptotic process may facilitate autoantibodies production that recognizes surface epitopes, mainly composed of phospholipid and β2 glycoprotein I for the removal of dying cells.

As mentioned earlier, the strength of this study was that we enrolled age- and sex-matched controls to control the confounders attributed to individuals who had different ages or sex [27–29]. Furthermore, we prospectively followed all participants to determine the association of aPL positivity and the risk of VTE/ATE or mortality in the study population. The limitations of this study were that we did not have enough power to demonstrate the differences between the subtype of aPL positivity and the risk of thrombosis. Secondly, all participants in our study were Asians. Consequently, the results of this study may not be generalized to patients with other ethnic groups who may have a higher risk of developing thromboembolic events. Furthermore, this study was conducted during the COVID-19 pandemic. We did not exclude the participants with a remote history of COVID-19 infection from the study due to limited evidence regarding the relationship between the infection and aPL positivity during the research design and planning period. However, cancer patients who have a positive test for COVID-19 would not be able to receive chemotherapy, according to the hospital policy at that time. As a result, those patients would not be enrolled in the study.

## Conclusion

Antiphospholipid antibodies (aPL) were more prevalent in ambulatory cancer patients than in non-cancer patients. Positive aPL in cancer patients was associated with an increased risk of composite outcome of VTE or ATE. The role of screening for aPL in cancer patients and prophylactic anticoagulation in further studies are warranted.

## Conflicts of interest

All authors declare no competing financial interests. C.K., P.N., A.T., L.N., E.R., T.R., S.H., P.P., T.P., N.H., and C.C. all declare no conflict of interest. No other potential conflicts of interest related to this study were reported. This study received research funding from the Faculty of Medicine, Chiang Mai University. All authors received salaries from the Faculty of Medicine, Chiang Mai University. However, the funder had no role in study design, data collection, analysis, decision to publish, or manuscript preparation.
